# On the Potential of Using Dual-Function Hydrogels for Brackish Water Desalination

**DOI:** 10.3390/polym10060567

**Published:** 2018-05-23

**Authors:** Wael Ali, Beate Gebert, Sedakat Altinpinar, Thomas Mayer-Gall, Mathias Ulbricht, Jochen S. Gutmann, Karlheinz Graf

**Affiliations:** 1 Physikalische Chemie and CENIDE (Center for Nanointegration), Universität Duisburg-Essen, Universitätsstr. 2, 45141 Essen, Germany; wael.ali@stud.uni-due.de (W.A.); sedakat.altinpinar@gmail.com (S.A.); mayer-gall@dtnw.de (T.M.-G.); 2 Deutsches Textilforschungszentrum Nord-West gGmbH, Adlerstr. 1, 47798 Krefeld, Germany; beate.gebert@dtnw.de; 3 Technische Chemie II and CENIDE (Center for Nanointegration), Universität Duisburg-Essen, Universitätsstr. 5, 45141 Essen, Germany; mathias.ulbricht@uni-due.de; 4 Physikalische Chemie, Hochschule Niederrhein, Adlerstr. 32, 47798 Krefeld, Germany; karlheinz.graf@hs-niederrhein.de

**Keywords:** hydrogel, desalination, thermally responsive polymer, polyelectrolyte, brackish water, *N*-isopropylacrylamide, acrylic acid, RAFT polymerisation

## Abstract

Although current desalination technologies are mature enough and advanced, the shortage of freshwater is still considered as one of the most pressing global issues. Therefore, there is a strong incentive to explore and investigate new potential methods with low energy consumption. We have previously reported that reversible thermally induced sorption/desorption process using polymeric hydrogels hold promise for water desalination with further development. In order to develop a more effective hydrogels architecture, polyelectrolyte moieties were introduced in this work as pendent chains and a thermally responsive polymer as network backbone using reversible addition-fragmentation chain transfer (RAFT) polymerisation. The ability of the comb-type polymeric hydrogels to desalinate water was evaluated. These hydrogels were proved to absorb water with low salinity from brine solution of 2 g L${}^{-1}$ NaCl and release the absorbed water at relatively low temperature conditions of 50 ${}^{\circ }$C. The fraction of the grafted polyacrylic acid and the comb-chain length were varied to understand their influence on the swelling/deswelling behaviour for these hydrogels. The ionic fraction in the hydrogels and the resulting hydrophilic/hydrophobic balance are crucial for the proposed desalination process. In contrast, the comb-chain length impacted the swelling behaviour of hydrogels but showed relatively little influence on the dewatering process.

## 1. Introduction

**Figure 1:**
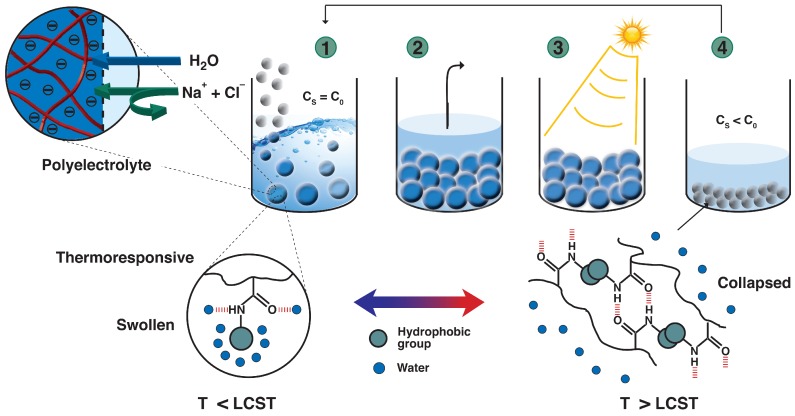
Scheme of the envisioned four steps of the desalination process, where (1) is swelling of the hydrogel in an excess of brine solution with concentration $C_{S}$ at which the mobile ions of the brine solution are partly rejected from the hydrogel (*left*), (2) removal of the excess brine solution, (3) dewatering of the hydrogel by means of a thermal energy source, the water is released from the hydrogel due to the phase separation of PNIPAAm above its LCST (*down*), (4) collection of salt depleted water with brine concentration less than initial one ( $C_{S}\;<\;C_{0}$ ).

Loosely cross-linked hydrophilic polymers that swell in the presence of water are often used to form jelly-like material known as hydrogels [1,2]. These types of materials offer a whole series of interesting potential applications not only because of their network structure and hydrophilic nature, but also due to their ability to respond to external stimuli such as temperature, pH, ionic strength, specific ions or molecules, electric field, magnetic field, and light [3,4,5,6,7]. For that reason, they are also known as smart or intelligent materials. On a molecular level, the swelling of the hydrogel networks in water results from the presence of hydrophilic chemical moieties. In particular, carboxylic (–COOH) groups attached to the polymer backbone or side chains enable the hydrogel to entrap a large amount of water due to the large swelling pressure caused primarily by charges of the carboxylate groups. Thermodynamically, the swelling of hydrogels is an osmotic process, where the water is transported from a region of higher water chemical potential to a region of a lower one [8]. Osmotic pressure is the driving force for many applications such as forward osmosis (FO) desalination in which a selectively permeable membrane allows passage of water, but rejects the solute molecules or ions despite a difference in solute concentration across the membrane [9]. In a similar manner, hydrogels built from polyelectrolytes in contact with brine will take up water with low salinity until the total change in free energy reaches a minimum, i.e., equilibrium between the two phases (inside and outside the gel) is reached. In this context, Höpfner et al. recently studied this effect in more detail and described a novel approach for water desalination using charged hydrogels under externally applied mechanical forces [10,11]. Here, we propose instead to use a temperature-sensitive polymer to desalinate salt water by means of reversible thermally induced absorption and desorption as illustrated schematically in Figure 1.

One of the most frequently used and studied temperature-responsive polymers and hydrogels is poly(*N*-isopropylacrylamide) (PNIPAAm), which demonstrates a sharp phase transition at a lower critical solution temperature (LCST) around 32 ${}^{\circ }$C in aqueous solution [12]. This is attributed to the changes in the local environment around the hydrophobic isopropyl domains below and above the LCST. The structured water layer around the hydrophobic regions lead to a negative entropy change of mixing, $\Delta S_{mix}$. Simultaneously, the binding of the water molecules to the polar amide groups causes a negative enthalpy of mixing, $\Delta H_{mix}$. According to the definition of the Gibbs free energy: $\Delta G_{mix}\;=\;\Delta H_{mix}\;-\;T\;\Delta S_{mix}$, raising the temperature above a certain value causes the entropy contribution ($T\;\Delta S_{mix}$) to exceed the enthalpy term ($\Delta H_{mix}$) resulting in a positive $\Delta G_{mix}$ or demixing [13]. Indeed, above the LCST, PNIPAAm chains adopt a coil-globule conformation, causing the hydrogel to undergo phase separation and collapse.

Recently, responsive polymer hydrogels have been utilised as a new class of draw agents for FO processes, and a range of parameters affecting the performance of this draw agent has been investigated [14,15]. In contrast, we used hydrogels as both membrane and drawing agent at the same time. Thus, the problem associated with a water flux has been circumvented because the interface is the hydrogel surface itself. Such a method can be used more efficiently because no additional equipment is needed. Moreover, the three-dimensional network of hydrogels with a tunable mesh enables them to act as a porous structure, which is very relevant for separation, segregation, and even filtration. Hence, such hydrogels can be expected to keep off contaminants via size selective exclusion or by charge-based selectivity (Donnan exclusion) even more easily [16,17,18,19,20]. At least their release from the gel, if there is any absorption in the gel by accident, could be retarded. On the other hand, higher charged ions often are subject to bridging forces [21], and therefore, some incorporation if such ions within the gel appear to be possible. This would offer the possibility to trigger controlled release of valuable ions as Ca^2+^ into the drinking water during the consecutive swelling/deswelling cycles. However, the challenge to combine an improved salt rejection with an efficient dewatering needs to be addressed.

In our previous work [22], hydrogels synthesised from sodium acrylate (SA) and the thermoresponsive co-monomer NIPAAm were shown to deplete the salinity of 2 g L${}^{-1}$ NaCl solutions by 23% in one cycle. Furthermore, the macromolecular architecture of the network structure affects the swelling/deswelling behaviour for those hydrogels. However, only 10% water was recovered from the swollen hydrogels under low temperature heating (50 ${}^{\circ }$C), as SA was mainly part of the backbone in the polymer network. Based on these results, we set out to study an optimised hydrogel architecture to increase water recovery. We modified the architecture on molecular level by synthesis of comb-type side chains where the polyelectrolyte moieties will be grafted as pendent chains on the thermoreversible backbone to introduce a force via collapse. We expect this change in macromolecular architecture to enhance the strong hydrophobic aggregation of PNIPAAm during shrinking, and thus increasing the release of water [23].

From a kinetic point of view, the slow growth rate in a living mechanism allows a polymer chain to be fully relaxed. Hence, the intermolecular cross-linking will be greatly increased compared to intramolecular cross-linking and microgel formation, resulting in branched and hyper-branched structures [24]. Furthermore, owing to the presence of freely mobile dangling chains in the network structure, which create more diffusion channels, the hydrogels can be expected to exhibit accelerated shrinking kinetics.

In the present study, we will discuss the preparation of comb-type polymer hydrogels composed of NIPAAm and a copolymer of polyacrylic acid (PAAc) via a two-step RAFT polymerisation, and verify their viability for water desalination. The influence of the length and the fraction of the grafted PAAc chains on the response rate and the equilibrium-swelling ratio will be investigated. Towards this end, three trithiocarbonate macro-RAFT agents (PAAc-TTC) differing in the average molecular weight ($M_{n}$) of the PAAc chain have been synthesised. The final grafted polymer networks (PNIPAAm-*g*-PAAc) were obtained after a second RAFT polymerisation using NIPAAm, PAAc-TTC, and cross-linkers with varying composition.

## 2. Materials and Methods

### 2.1. Materials

*N*-Isopropylacrylamide (NIPAAm, Sigma-Aldrich, St. Louis, MO, USA, >99%) was purified by recrystallisation in hexane and dried in vacuo. Acrylic acid (AAc, Sigma-Aldrich, >99%) was vacuum-distilled to remove the inhibitor MEHQ (hydroquinone monomethyl ether) and possible oligomer content. 4,4${}^{\prime }$-Azobis-4-cyanopentanoic acid (ACPA, 98%), *N*,*N*${}^{\prime }$-methylenbisacrylamide (MBAm, 99%), 2-(dodecylthiocarbonothioylthio)-2-methylpropionic acid (TTCA, 98%), 1,4-dioxane (anhydrous, 99.8%), 1,3,5-trioxane (>99%), ethanol (99.8%) were all purchased from Sigma-Aldrich and used as received. Sodium chloride (99.5%) and diethyl ether (99%) were obtained from Acros Organics, Geel, Belgium. The water used throughout this study was deionised water from a Milli-Q system (Millipore, Burlington, MA, USA).

### 2.2. Preparation of Hydrogels

#### 2.2.1. Synthesis of the Poly(acrylic acid)macromolecular RAFT Agents (PAAc-TTC)

The poly(acrylic acid)-based macro-RAFT agent was prepared similarly as described elsewhere [25]. In a typical experiment (Table 1, entry E2), AAc (6.100 g, 84.50 mmol), ACPA (18.30 mg, 0.065 mmol) as an initiator, TTCA (237.0 mg, 0.650 mmol) as a RAFT agent and 1,3,5-trioxane (350.5 mg, 3.891 mmol) as an internal reference for Nuclear Magnetic Resonance (NMR) analysis were charged to a three-necked flask with a magnetic stirrer and dissolved in 1,4-dioxane (35 mL). The reaction solution was purged for 20 min with nitrogen and then degassed by a freeze-thaw cycle and sealed under nitrogen. The polymerisation was carried out at 70 ${}^{\circ }$C in a thermostated oil bath under stirring and stopped after 80 min at 48.8% of monomer conversion (the overall monomer conversion was determined by ^1^H-NMR spectroscopy in $d^{6}$-DMSO by the relative integration of the internal reference peak at 5.10 ppm and the vinylic proton peaks of AAc at 5.90, 6.08, and 6.31 ppm). The polymer was recovered by precipitation in cold diethyl ether and then dried under reduced pressure overnight.

#### 2.2.2. Synthesis of Comb-Type Grafted PNIPAAm-*g*-PAAc Networks

To synthesise the comb-type grafted gels (GG), a mixture of PAAc-TTC, NIPAAm, MBAm, ACPA were dissolved in 3 mL ethanol and the solution was charged to a glass vial (10 mm in diameter) equipped with a magnetic stirrer. The solution was purged over a period of 20 min with nitrogen and then degassed by a freeze-thaw cycle and sealed under nitrogen. The polymerisation was conducted for at least 72 h at 70 ${}^{\circ }$C. The obtained gels were immersed in ethanol for 5 days and in deionised water for another 7 days at room temperature with solvent being daily refreshed to remove unreacted materials and allowing equilibrium swelling. Then, gels were dried under ambient conditions for 5 days. The conversions of the monomer were calculated according to the following equation:

(1) $$Conversion\;\left(wt\;\%\right)\;=\;\frac{m_{gel}}{m_{PAAc-TTC}\;+\;m_{NIPAm}\;+\;m_{MBAm}\;}\;\times \;100$$

A comprehensive account on the samples used and their respective composition as well as the degree of monomer conversions are given in Table 2.

### 2.3. Characterisation and Measurements

#### 2.3.1. Scanning Electron Microscopy (SEM)

The morphology of hydrogels were examined using scanning electron microscopy (S-3400 N II, Hitachi High-Technologies Europe GmbH, Tokyo, Japan). The hydrogel specimens were firstly immersed in deionised water at room temperature (23 ${}^{\circ }$C) until a maximum swelling is achieved. The hydrogels were subsequently frozen at −196 ${}^{\circ }$C in liquid nitrogen. The frozen samples were then fractured and freeze-dried at −50 ${}^{\circ }$C for 24 h using a freeze dryer system (Christ Alpha 1-4, Osterode, NI, Germany). The fractured surface of the samples were sputtered with gold in vacuum for 4 min using a Quorum Emitech K500X sputter coater (Ashford, Kent, UK).

#### 2.3.2. Nuclear Magnetic Resonance Spectroscopy (NMR)

${}^{1}$H-NMR spectra of macro-RAFT agents were recorded on a Bruker DMX-300 instrument (Bremen, HB, Germany) at 300 MHz using deuterated dimethyl sulfoxide as solvent. The spectra were also used to determine the conversion of monomer and the average molecular weight of the macro-RAFT agents.

#### 2.3.3. Fourier-Transform Infrared Spectroscopy (FTIR)

FTIR spectra of the macro-RAFT agents (PAAc-TTC) and hydrogels were recorded on a 3100 Fourier-Transform infrared spectrometer (Varian, now Agilent Technologies, Santa Clara, CA, USA) in the range of 600–4000 cm${}^{-1}$ with an average of 32 scans and a resolution of ±4.0 cm${}^{-1}$. All measurements were performed using the attenuated total reflectance (ATR) mode.

#### 2.3.4. Differential Scanning Calorimetry (DSC)

The water content was estimated using differential scanning calorimetry (DSC) (Netzsch 204 Phoenix, Selb, BY, Germany) from −30 to 30 ${}^{\circ }$C. The hydrogels were allowed to swell in deionised water to equilibrium at room temperature prior to DSC measurements. Hydrogel samples (∼5 mg for each one) were encapsulated in a hermetically sealed aluminium pan and cooled to −40 ${}^{\circ }$C using liquid nitrogen where the temperature was then held iso- thermally for 5 min. The sample was then heated at a heating rate of 3 ${}^{\circ }$C min${}^{-1}$ under nitrogen atmosphere. The presence of free and bound water was estimated by following a standard procedure described by Mansor and Malcolm [26]. The amount of bound water was calculated, assuming that the heat of fusion of unbound water inside the hydrogel is the same as that for ice applying the following equation:

(2) $$W_{b}\;\left(\%\right)\;=\;W_{eq}\;-\;\left(W_{f}\;+\;W_{fb}\;\right)\;=\;W_{eq}\;-\;\left(\frac{Q_{endo}}{Q_{pure}\;}\right)\;\times \;\;100$$

where $W_{eq}$ is the equilibrium water content (EWC) (%), $W_{b}$, $W_{f}$, and $W_{fb}$ are the amount of non-freezable bound water (%), freezable free water (%) and freezable bound water (%), respectively, whereas $Q_{endo}$ is the heat of fusion of freezable water (freezable bound water and freezable free water) within the hydrogels. $Q_{pure}$ is the heat of fusion of ice (340.1 J g${}^{-1}$). The amount of unbound water was then calculated by the subtracting the amount of bound water from the equilibrium water content.

#### 2.3.5. Swelling Ratio and Kinetics of Swelling

The hydrogel samples were placed in excess water with a typical mass ratio of 0.05 ±0.01 gel/water. The swelling ratios ($SR_{eq}$) of the hydrogels were gravimetrically measured after removing the excess surface water with a moistened filter paper in the temperature range from 15 to 50 ${}^{\circ }$C for 24 h at each individual temperature. Swelling ratio and equilibrium water content (EWC %) of polymer hydrogels were defined as follows:

(3) $$SR_{eq}\;=\;\frac{W_{s}\;-\;W_{d}}{W_{d}\;}\;$$

(4) $$EWC\;\left(\%\right)\;=\;\left(\frac{W_{s}\;-\;W_{d}}{W_{s}\;}\;\right)\;\times \;\;100$$

where $W_{s}$ is the weight of the swollen hydrogels at room temperature and $W_{d}$ is the weight of the hydrogels dried at ambient conditions. The swelling values at equilibrium were taken after 24 h in both salt solutions of 2 g L${}^{-1}$ NaCl and in deionised water (DI). Quantitative estimation of the swelling kinetics for the hydrogels were also obtained by measuring the weight changes of gels in deionised water at regular time intervals after water was wiped from the gels using filter paper. The water uptake (WU%) at regular time intervals was defined as follows:

(5) $$WU\;\left(\%\right)\;=\;\left(\frac{W_{t}\;-\;W_{d}}{W_{s}\;-\;W_{d}\;}\;\right)\;\times \;\;100$$

where $W_{t}$ is the weight of the gel at each particular time. To analyse the swelling process quantitatively, half-swelling time ($t_{1/2}$) was also defined, at which 50% water uptake occurred.

#### 2.3.6. Determination of Water Recovery and Salt Rejection

The dewatering experiments were carried out at 50 ${}^{\circ }$C for 60 min. The weight difference of the hydrogels before and after the dewatering test was calculated to determine the amount of water released from hydrogels. The water recovery was then derived as follows:

(6) $$Water\;recovery\;\left(\%\right)\;=\;\left(\;\frac{W_{R}\;}{W_{s}\;-\;W_{d}\;}\;\right)\;\times \;\;100$$

where $W_{R}$ is the weight of water released in the dewatering test.

The salt rejection was obtained by measuring the concentrations of sodium ions in the water recovered from the hydrogels by ion chromatography (Metrohm 833 Basic IC plus). Silica gel with carboxyl groups column (particle size 5 mm) was used at 25 ${}^{\circ }$C. The eluent was (1.7 mmol L${}^{-1}$ nitric acid + 0.7 mmol L${}^{-1}$ dipicolinic acid) at a flow rate of 0.9 mL min${}^{-1}$. The chromatograph was calibrated with a standard solution of Na^+^. The salt rejection was determined as follows:

(7) $$Salt\;rejection\;\left(\%\right)\;=\;\left(\;1\;-\;\frac{C_{R}\;}{C_{0}\;}\;\right)\;\times \;\;100$$

where $C_{R}$, $C_{0}$ are the sodium concentrations of the water recovered from the hydrogels and that of the feed solution (2 g L${}^{-1}$ NaCl), respectively.

#### 2.3.7. Rheological Measurement

The mechanical properties of the fully swollen hydrogels were investigated via the frequency sweep experiments using a rheometer (Anton Paar, Graz, Austria, model MCR-300). Solvent trap was used to minimize evaporation during the measurments. The experiments were performed in the linear range of ω = 0.1–100 rad s${}^{-1}$ with a fixed strain of 1% and a normal force (FN) of 1 N and at 20 ${}^{\circ }$C using a 15 mm diameter TruGap plate corrected with a “true gap” function of the instrument.

#### 2.3.8. Mesh Size Calculation of Hydrogels

Based on the rheological data and the theory of rubber elasticity [27], the values of storage modulus at infinitesimal deformations were used to estimate the mesh size of the bulk hydrogels applying the following relationship:

(8) $$G^{\;\prime }\;=\;n_{cl}\;R\;T$$

where $G^{\;\prime }$ is the value of storage modulus at plateau region measured with frequency sweep experiment, $n_{cl}$ is the number density of elastically effective cross-linking points (mol m${}^{-3}$), *R* is the ideal gas constant (J mol${}^{-1}$ K${}^{-1}$), and *T* the measuring temperature in *K*. Thus, at a given temperature, an increase in the number of network junctions resulting in a proportional increase in the steady-state value of the storage modulus. The elastic term obtained from rheological measurement is then associated to the mesh size of the hydrogel network with the following equation [28]:

(9) $$L\;=\;\xi \;=\;{\left(\;\frac{1}{n_{cl}\;N_{A}}\;\right)}^{1/3}\;=\;{\left(\;\frac{R\;T}{G{\;}^{\prime }\;N_{A}}\;\right)}^{1/3}$$

where $N_{A}$ is Avogadro’s constant with the assumption that the clusters are evenly dispersed and each cluster is positioned in the centre of a cubic-shaped volume element. Thus, the side length *L* of any cubic element can be calculated with Equation (9) since all cubic elements together span the total gel.

## 3. Results and Discussion

### 3.1. Synthesis of Hydrogels

**Scheme 1:**
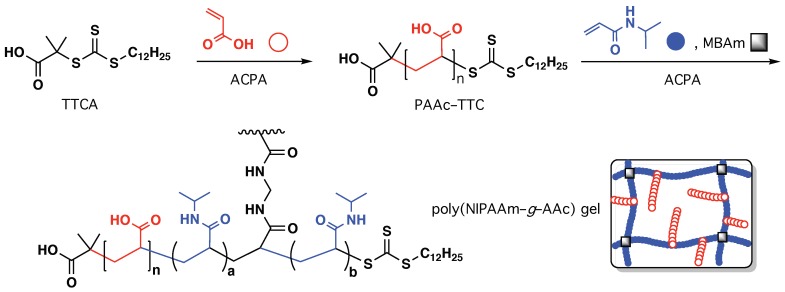
Synthesis of the PNIPAAm-g-PAAc hydrogel network.

The incorporation of ionisable functional groups such as AAc into PNIPAAm-based gels via random copolymerisation significantly affects the temperature-responsive behaviour and hence their volume changes. Therefore, a graft structure with PAAc freely mobile chains could maintain the PNIPAAm chain aggregation without interfering with the large amount of ionic moiety in the network. To achieve this macromolecular architecture, comb-type grafted hydrogels were prepared by RAFT polymerisation. The synthesis route is shown in Scheme 1. The PAAc-TTC macro-RAFT agents were initially prepared by homopolymerisation of AAc in the presence of TTCA as chain transfer agent using 1,4-dioxane as reaction medium, which exhibits low chain-transfer activity. The polymerisations were stopped at low monomer conversion in order to recover a large proportion of trithiocarbonate-terminated chains. The resulting macro-RAFT agents were characterised by ^1^H-NMR and FTIR (see Table 1 and Figures S1 and S2 in Supplementary Materials).

The PAAc-grafted PNIPAAm networks were then obtained via a second RAFT polymerisation using three PAAc-TTC (differing in their chain length) as macro-RAFT agent and MBAm as cross-linker agent (Table 2). The reaction mixture gradually gelled as the polymerisation proceeded and the onset as well as the end of gelation were delayed with increasing the chain length of the macro-RAFT agent. Under the present conditions, the maximum conversion of NIPAAm was as high as 90% and decreased with an increase of the content of the macromolecular RAFT agent. Furthermore, no gels were formed with PAAc content higher than 40 wt %, which we attribute to the formation of hyperbranched copolymers of NIPAAm and MBAm (cf. Table 2) [29]. It should also be noted that gels were formed even in the absence of a cross-linker when the polymerisation was carried out in 1,4-dioxane, which can be attributed to the formation of hydrogen-bonded complexes [23]. Many solvents have been tested to avoid such behaviour and we found that absolute ethanol was a suitable solvent for our system [30].

### 3.2. Characterisation of Hydrogels

The polymer hydrogels were characterised with FTIR (Figure S3 in Supplementary Materials). All spectra exhibit a characteristic peak at 1711 cm${}^{-1}$ assigned to the stretching vibration of C=O bonds of carboxylate groups of PAAc, and two absorbance bands at 1624 cm${}^{-1}$ and 1540 cm${}^{-1}$ attributed to the secondary amide stretching of PNIPAAm (aka amide I and II bonds, respectively). The broad bands in the range of 3100–3600 cm${}^{-1}$ are due to the N–H stretching, while a C–N stretching band appears at ∼1455 cm${}^{-1}$, confirming the presence of amide groups.

**Figure 2:**
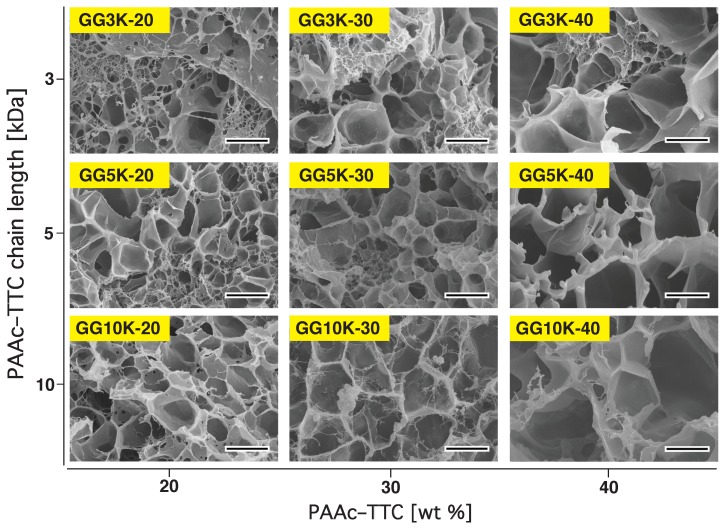
SEM images of the freeze-dried hydrogels with scale bars of 5 μm.

The morphological features of the swollen freeze-dried hydrogels were evaluated via SEM studies. The micrographs shown in Figure 2 reveals that all gels exhibit a heterogeneous interconnected porous-like structure. During the freeze-drying process, the formation of ice crystals within the network upon immersing the samples into liquid nitrogen acts as a template for pore generation resulting in macropores structure [31,32]. Therefore, this observed morphology is compact aggregates of the polymer chains rather than the mesh of hydrogels at their molecular level. Nevertheless, the macrostructure of freeze-dried hydrogels depends remarkably on the PAAc-TTC concentration. With higher PAAc content, the grafted hydrogel samples show larger internal pores due to the lower polymer fraction in the hydrogel that corresponds to higher water content. In contrast, the chain length of the PAAc appears to have a minor influence on the porous structure.

#### 3.2.1. Swelling Properties

**Figure 3:**
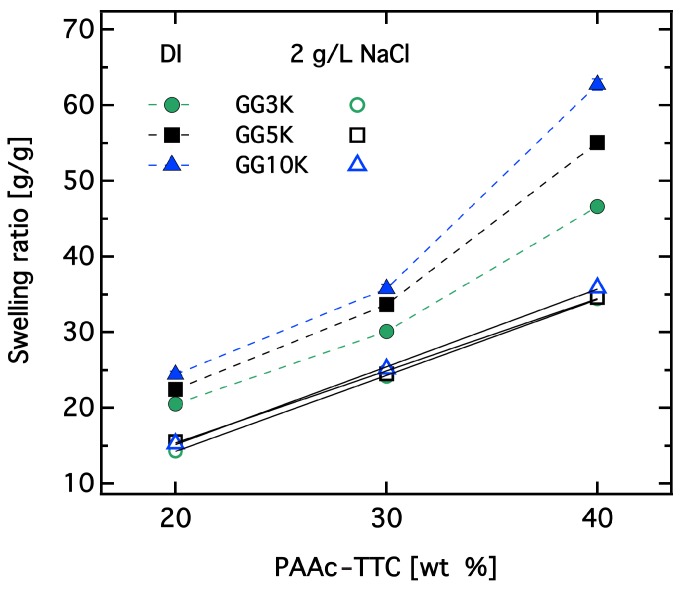
Equilibrium swelling ratios ($SR_{eq}$) of the hydrogels in deionised water (DI) (*closed symbols*) and in salt solution (2 g L${}^{-1}$ NaCl) (*open symbols*) as a function of the PAAc content at room temperature (∼23 ${}^{\circ }$C). The dashed lines are just a guide for the eye while the straight ones are linear fits.

The swelling behaviour of the comb-type grafted PAAc gels was investigated in both brine and deionised (DI) water. It is well known that the swelling and osmotic pressure within the hydrogel increases with increasing the ionic component and thus the equilibrium-swelling ratio of the hydrogel will be increased. Figure 3 shows that indeed the equilibrium-swelling ratio of all synthesised hydrogels increases with increasing the PAAc content. The length of grafted chains also affects the swelling behaviour of the hydrogel. Therefore, the gels with longer PAAc chains show higher equilibrium-swelling ratios as shown in Figure 3.

The strong hydration of the gels is related to the fact that longer chains are structurally separated from the backbone of the cross-linked network. The hydrogels with different PAAc-grafted chain lengths may have different cross-linking density. The effect of a change in crosslink density on the equilibrium-swelling ratios was not studied. No effect of the chain length on the equilibrium-swelling ratio in brine was found indicating that the contribution of charges to the osmotic pressure is the same in all systems. We therefore expect samples containing the same total amount of PAAc to show a similar salt depletion capacity.

**Figure 4:**
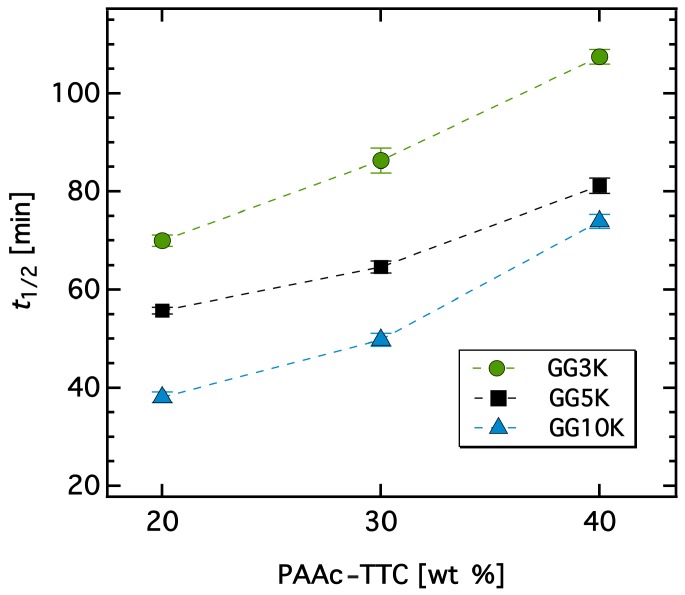
Plots of half-swelling time ($t_{1/2}$) as a function of the PAAc content. The dynamic study was evaluated in deionised water (DI) at room temperature (∼23 ${}^{\circ }$C). The shape and size of the cylindric hydrogel samples were constant ( 5 mm in diameter and 2 mm in thickness).

The dynamic of the swelling process of the dry materials was also quantified via the half-swelling time ($t_{1/2}$), at which 50% water was taken up (Figure S4 in Supplementary Materials). Generally, the swelling process can be described by three transition steps [33]: the diffusion of water into the glassy polymer matrix, the transition of polymer phase from glassy state to rubbery state (due to polymer hydration), and the collective diffusion of polymer network toward the surrounding water. Figure 4 shows that the effect of both the graft chain amount and the length on the swelling kinetics was evident. The lower the content of PAAc in the hydrogel was, the faster was the water uptake. Gels with higher grafted PAAc content fill more voids in the hydrogel network and this leads to more compact structure. Hence, the transition of polymeric materials from glassy to rubbery state will be slower. The other factor, which may hinder the diffusion of water into the hydrogels, is the hydrogen bonding interactions between the grafted PAAc chains and the PNIPAAm backbone. In contrast, at a fixed amount of grafted PAAc chains, gels with a longer chain length undergoes faster hydration rate. This contradictory behaviour can be attributed to the faster collective diffusion of water in systems with longer polymer chain length upon the transition of the polymer phase from glass to rubbery state.

#### 3.2.2. Rheological Investigation and Network Structure

The mechanical properties of the fully swollen hydrogels in deionised water were characterised by measuring the influence of the angular frequency on elastic modulus ($G^{\;\prime }$) and loss modulus ($G^{\;\prime \prime }$) (Figure S5 in Supplementary Materials). All gels exhibit a plateau, i.e., frequency independent response of the storage modulus in the range from 0.1 to 100 rad s${}^{-1}$, which reveals that those gels indeed exhibit a soft rubbery-like behaviour. At higher frequencies, gels with a PAAc content of 40 wt % show a slight increase in $G^{\;\prime }$. This behaviour is most likely attributed to the more or less flexible grafted PAAc chains that exhibit longer relaxation times, i.e., they require lower applied frequencies to rearrange themselves on the time scale of the imposed mechanical motion resulting in a gradual rise of $G^{\;\prime }$.

**Figure 5:**
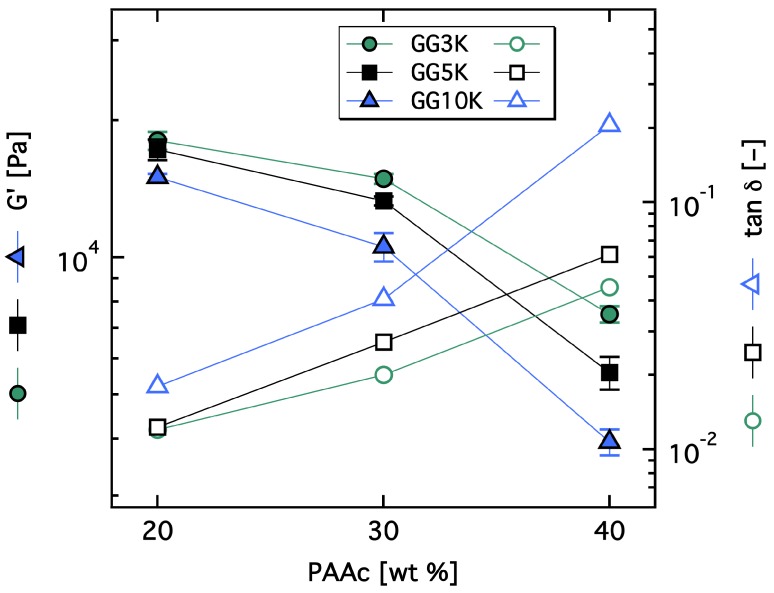
Storage modulus ($G^{\;\prime }$) and loss tangent (tanδ) as a function of grafted PAAc content of the fully swollen hydrogels.

**Figure 6:**
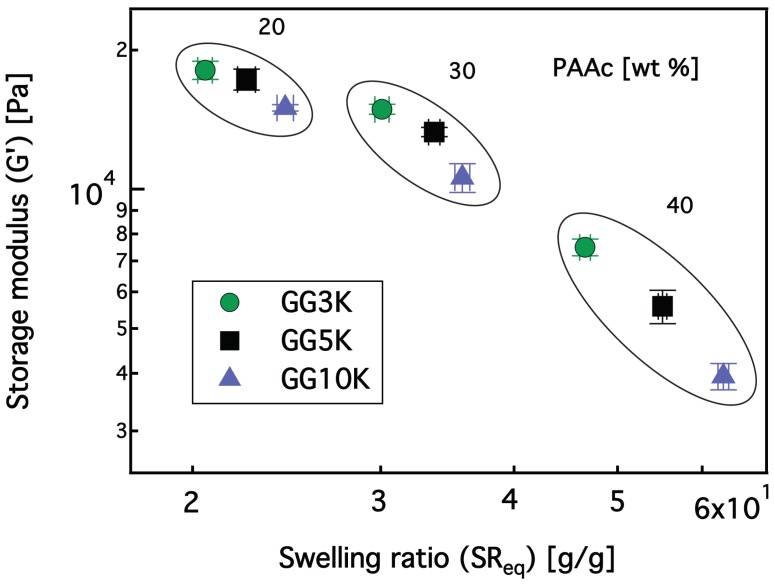
Storage modulus ($G^{\;\prime }$) as a function of the equilibrium swelling ratio of gels with different fraction of PAAc and different comb-chain lengths.

However, the change in modulus arise from entropy changes in the rubbery range. Therefore, the change in toughness or stiffness of the hydrogel networks can be correlated with their composition. Figure 5 shows that there is indeed an increase of $G^{\;\prime }$ with both the content and the length of the grafted PAAc chains. Gel networks with more flexible grafted PAAc chains exert a lower elastic force and thus a higher loss tangent due to the strong dependence on the water content of hydrogels at fully swollen state. As we stated regarding the swelling properties presented above, the graft chains’ mobility affects the gel swelling behaviour, namely the equilibrium swelling ratio. Therefore, a correlation between storage modulus and swelling ratio at equilibrium could be confirmed (Figure 6). Gels with more water per unit volume exhibit a reduction of the elastic modulus.

**Figure 7:**
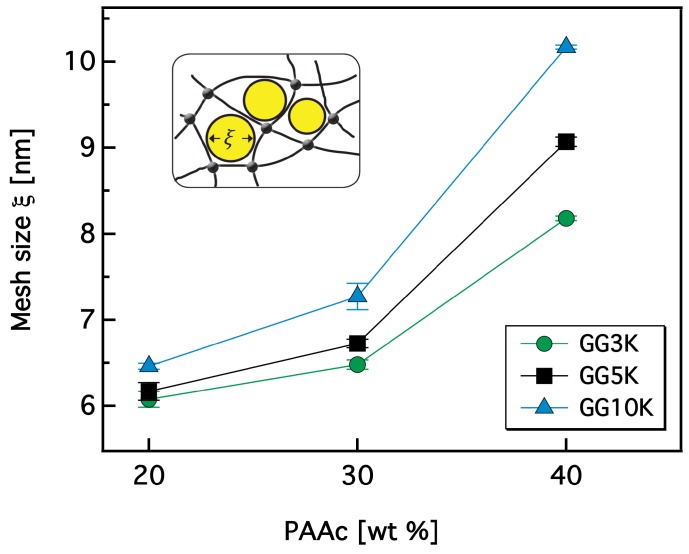
Correlation of the mesh size estimated from rheological data with the PAAc content of hydrogels in deionised water (DI).

The mesh size of hydrogels, as a measure of their microstructure, was estimated from the rheological data using Equation (9). As expected, the mesh size increases with increasing both, the PAAc content and the length of the grafted PAAc chains (Figure 7). This result is in agreement with the SEM observations and the swelling behaviour, where high PAAc content shows larger internal pores in SEM and more water in equilibrium-swelling compared with those with less PAAc content.

### 3.3. Dewatering Behaviour of Hydrogels and Salt Rejection

**Figure 8:**
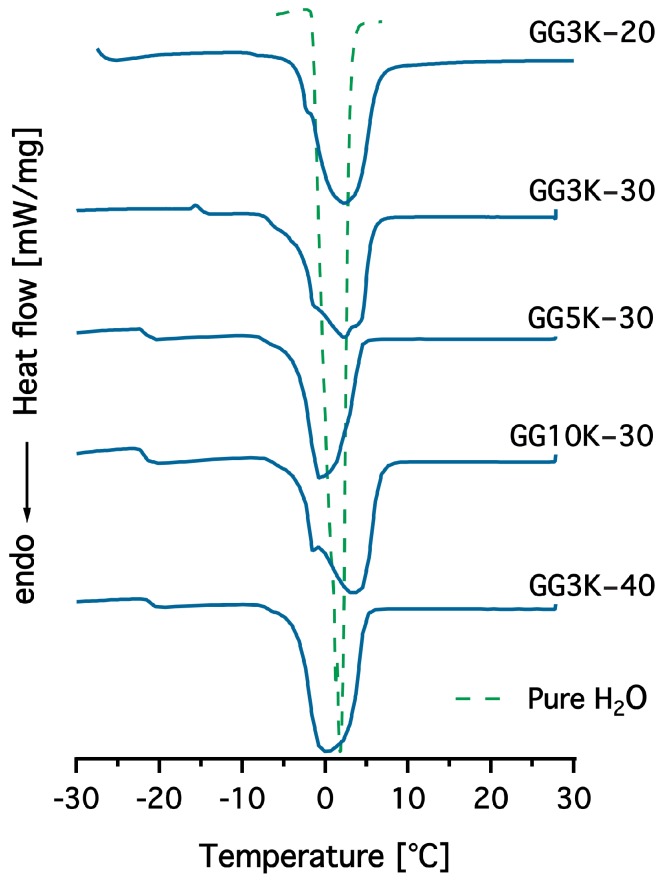
DSC thermograms of the fully swollen hydrogels from which the freezable water content was determined; water as reference.

In order to get an insight into the dewatering of gel matrices, the type of water binding inside the fully swollen hydrogels was determined by means of DSC analysis. Generally, water inside hydrogel networks can be thermodynamically classified as one of three different states [34]: (1) free water, which does not take part in hydrogen bonds with the polymer chains; this free interstitial water should be easily removed under high temperature conditions as it is physically entrapped within the polymer networks, and thus it behaves similarly to pure water as far as freezing and melting is concerned; (2) bound water, which directly binds to polymer chains via hydrogen bounding; this type of water is an integral part of the hydrogel structure and cannot easily be separated from it; thus it does not show any endothermic peak within the normal temperature range associated with pure water; and (3) semi-bound water, that exhibits weak interactions with polymeric chains within the network of hydrogels and freezes/melts at a temperature shifted with respect to that of free water. DSC thermograms of the fully swollen hydrogels in DI water (Figure 8) display that the melting of water in all samples starts at temperatures lower than that of pure water (*dashed line*). Generally, for all studied gels, the endothermic peaks are broad and structured.

**Figure 9:**
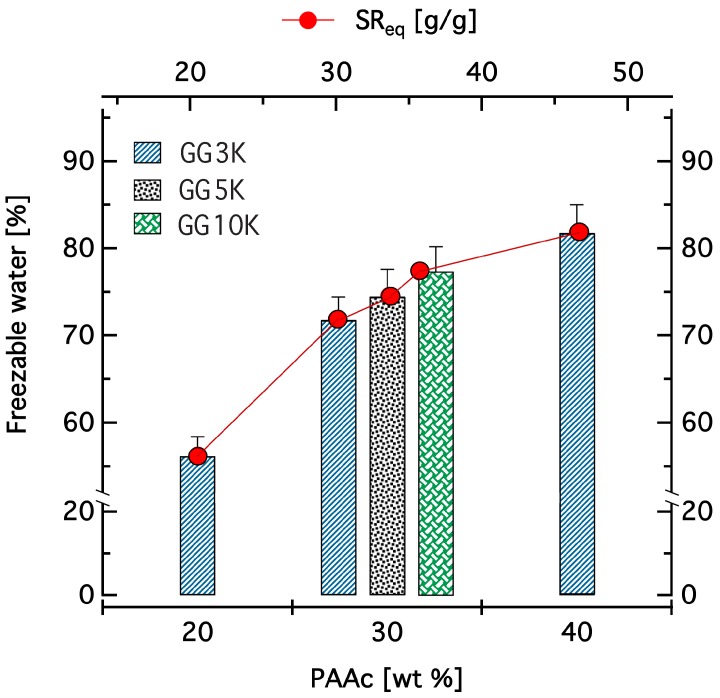
Freezable water content estimated from the DSC thermograms for the studied hydrogels as a function of grafted PAAc content (*bars mode*) and swelling ratio at equilibrium (*markers mode*).

The different fraction of freezable and non-freezable water for the studied hydrogels can be correlated with their swelling ratio at equilibrium, which is also associated with both grafted PAAc content and chain length. The grafted PAAc with freely mobile ends can hydrate sufficiently and contain a large amount of freezable water. Figure 9 shows that the fraction of freezable water increases gradually with increasing PAAc fraction in the hydrogel. A slight increase in the freezable water content was also observed for increasing grafted PAAc chain length. Accordingly, the different swelling ratios at equilibrium between the gels lead to a different freezable water content.

**Figure 10:**
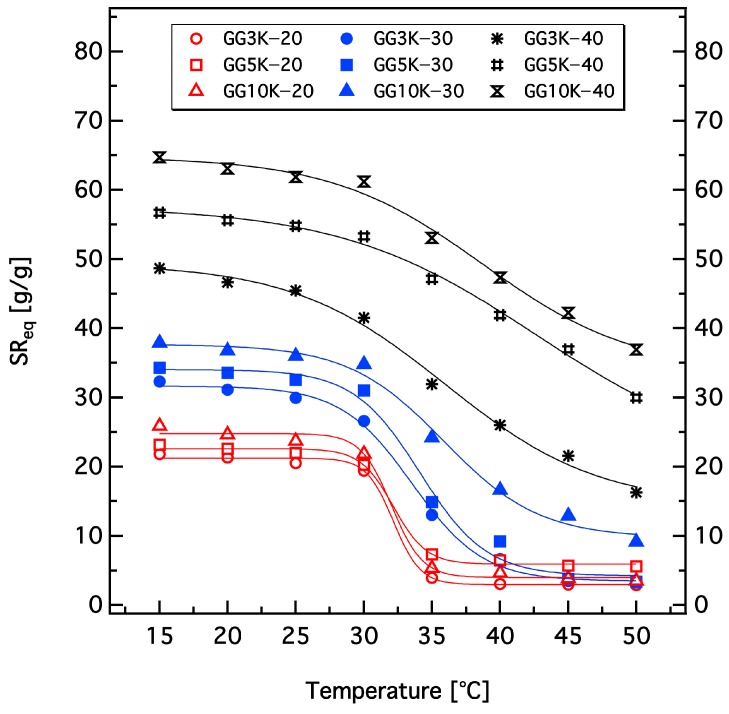
Equilibrium swelling ratio ($SR_{eq}$) of hydrogels as a function of temperature in deionised water. The lines are fit of a sigmoid function to the raw data.

As we stated in the Introduction, the solution taken up by gels should not only be depleted from salt, but also can swiftly be released under thermal stimulus. Therefore, the phase transition behaviour of hydrogels was explored by measuring the changes of the swelling ratios at equilibrium as a function of external temperature in deionised water as shown in Figure 10. All hydrogels displayed a sigmoid curve of swelling ratio versus temperature, where the gels swell at lower temperature and shrink above the LCST of PNIPAAm that occurred at around 32 ${}^{\circ }$C. This is because the hydrophobic interactions between the hydrophobic groups of the hydrogels become dominant and thus the gel matrixes shrinks. Optically, gels with 20 and 30 wt % PAAc changed their appearances from transparent at 15 ${}^{\circ }$C to opaque at 50 ${}^{\circ }$C while gels with 40 wt % PAAc kept their transparency. This may be attributed to the loose structure of the latter gels. Hence, the macropores of certain size still retain water even in a shrunken state, thus keeping the samples transparent. This would qualitatively also correlate with the SEM data for the frozen gels (cf. Figure 2).

The LCST at which the volume phase transition of the gels start is obtained by a fit of a sigmoidal function to the data (Figure 10). Gels with PAAc content of 20 wt % show a LCST at 32 ${}^{\circ }$C, while the phase transition temperature for gels with higher PAAc content increased up to 43 ${}^{\circ }$C. Moreover, the relative change of the swelling ratio or the percentage of released water in the temperature range of 30 to 35 ${}^{\circ }$C decreases and the transition itself becomes broader with increasing PAAc content and chain length. The highly hydrophilic nature of PAAc enhances the hydration of gels via a repulsive force between the strongly hydrated and charged carboxylate anions. However, the increased repulsion also restricts the hydrophobic network aggregation resulting in a distribution of LCST over a broader temperature range as the fraction of ionic moiety increases.

**Figure 11:**
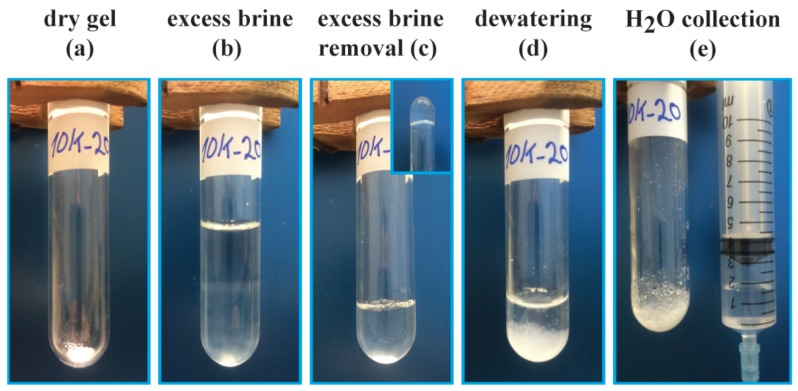
Photographs represent the steps of the desalination process using powder gel materials, where (**a**) is the gel in dry state; (**b**) swelling of the gel in excess salt water; (**c**) removal of the excess solution; (**d**) dewatering of the gel by thermal heating for 60 min at 50 ${}^{\circ }$C; (**e**) collection of the recovered water.

The water release from the polymer hydrogels (Figure 11) was achieved via external heating and the percentage of water recovery was determined after 60 min of dewatering at a relatively low temperature (50 ${}^{\circ }$C).

**Figure 12:**
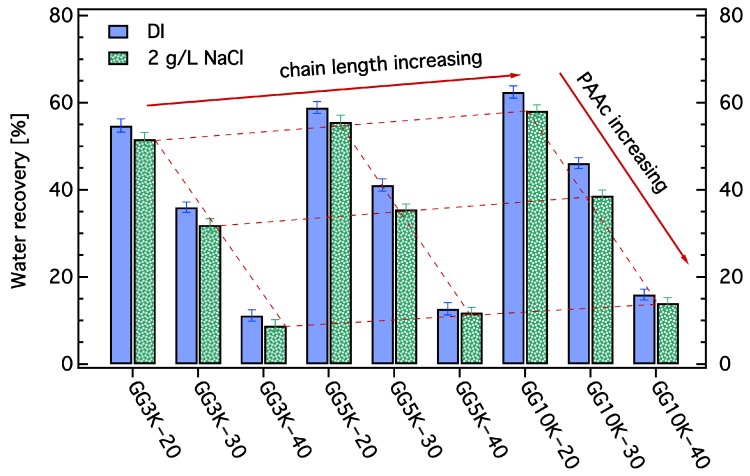
Percentage of recovered liquid water from fully swollen gels (powder form) in both deionised water (DI) and salt solution (2 g L${}^{-1}$ NaCl) after 60 min dewatering process at 50 ${}^{\circ }$C.

Figure 12 shows the percentage of water recovery from swollen gels with various grafted PAAc contents and chain length starting with powder after equilibrium loading in both deionised water (DI) and brine solution of 2 g L${}^{-1}$ NaCl. For all hydrogels, the water recovery decays dramatically as PAAc content increases. Hydrogels with 20 wt % of PAAc show the most powerful dewatering ability. The recovered liquid water fraction from the equilibrium swelling state was above 50%, while the water recovery for gels with 30 and 40 wt % of PAAc exhibit a reduction by a factor of 1.6 and 5, respectively. This is because the hydrophobic aggregation of the thermoresponsive moieties at the utilised temperature and on the time scale of the imposed dewatering process is restricted by the highly hydrated carboxylate groups that minimises the possibility of depletion processes.

In addition, gels with longer PAAc chain length have a slightly better dewatering performance, resulting probably from the increase in void volume within the polymer network upon the dehydration of PNIPAAm chains (in qualitative agreement with SEM data: cf. Figure 2). This is also supported by the slight dependence of the freezable water content with increasing chain length (cf. Figure 9), where the relative change is roughly the same. It is worth noting that faster dewatering responses were also observed for gels with longer grafted chains.

**Figure 13:**
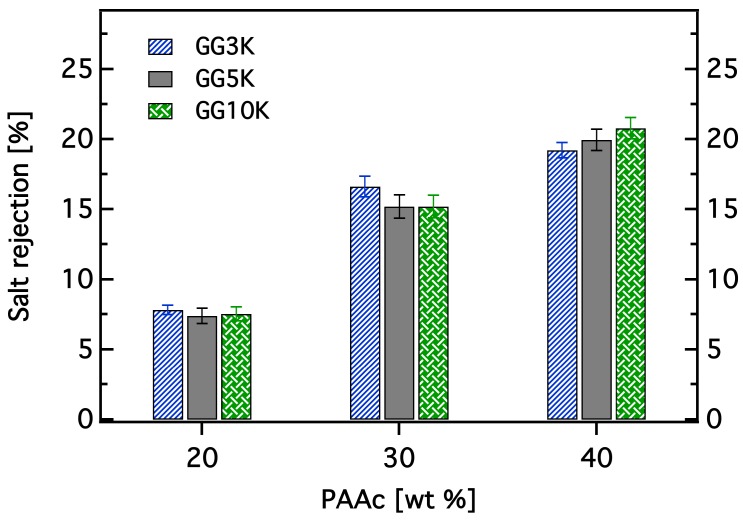
Salt rejection upon swelling/dewatering process of hydrogels in powder form with 2 g L${}^{-1}$ NaCl as feed saline water in one cycle.

The salt rejection was measured via the ion concentrations of the salt solutions before swelling and after 60 min dewatering for each hydrogel. The rejection of salinity increases with increasing ionic moiety fraction as shown in Figure 13. As we expected, no effect on the salt rejection has been observed with altering the PAAc-grafted chain length, since those hydrogels contain the same total fraction of ionic component. However, the salt rejection triples from 20 to 40 wt % PAAc content.

**Figure 14:**
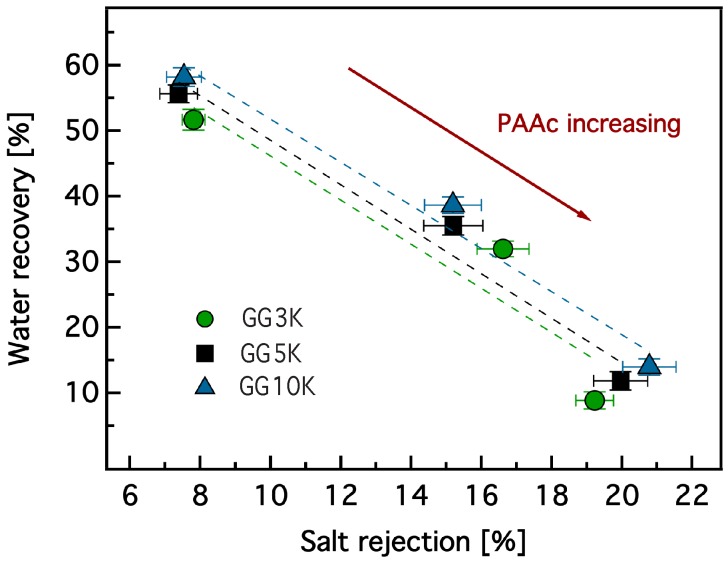
The competition between hydrophilic and hydrophobic balance on the performance of hydrogels in the proposed desalination process. The salt rejection increases as the water recovery decreases. Gel samples in powder form were swollen in excess salt solution of 2 g L${}^{-1}$ NaCl from the dry state and shrunk throughout 60 min at 50 ${}^{\circ }$C. The straight lines are linear fits to the data.

**Figure 15:**
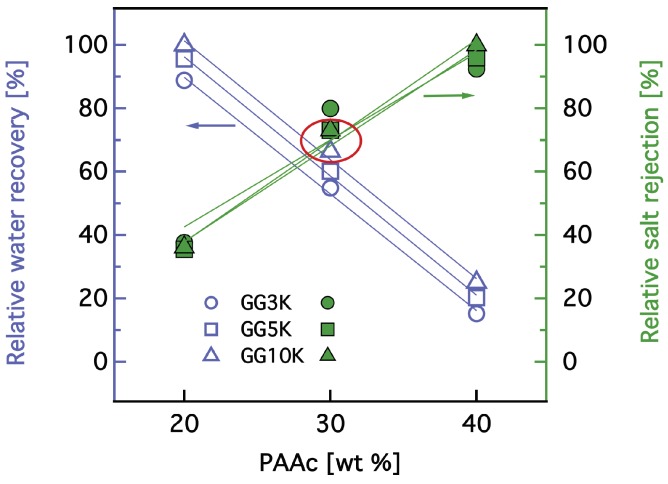
Overview of the performance of hydrogels in terms of desalinated water recovery and salt rejection as a function of grafted PAAc content. The straight lines are linear fits.

The opposing behaviour of gels to absorb water with less salinity and easily release it in the dewatering process is nicely illustrated when the water recovery is plotted versus the salt rejection (Figure 14). Increasing the hydrophilic volume fraction, i.e., the ability of salt rejection counteracts the water recovery that is triggered via the hydrophobic volume fraction of the hydrogel. Therefore, to predict the optimum performance of such materials as a function of the hydrophilic or the hydrophobic content, a relative percentage of both the salt rejection and the desalination capacity (water recovery) was separately calculated according to the best working materials. The obtained data were evaluated in terms of the intersection of the two linear functions as shown in Figure 15 or as a sum function of the two relative performances (Figure S6 in Supplementary Materials). Clearly find that the gel with 30 wt % of PAAc and longer chain length shows the best trade-off for such desalination experiments. It worth noting that, in the same experimental conditions, the water recovery of PNIPAAm gel does not exceed 70%. This is supported by the Donnan membrane theory according to which highly charged gels are expected to show the largest effect of salt depletion [35]. However, even pure polyacrylic acid gels could not achieve a high salt rejection, and their performance decreases as the initial brine concentration increases [10].

**Figure 16:**
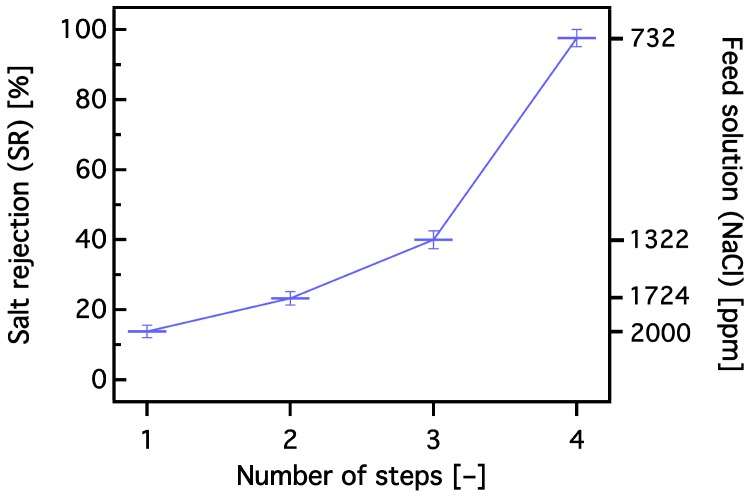
Total number of steps required to achieve a complete salt rejection (SR) using GG10k–30 hydrogel. The straight line is a guide for the eye.

**Figure 17:**
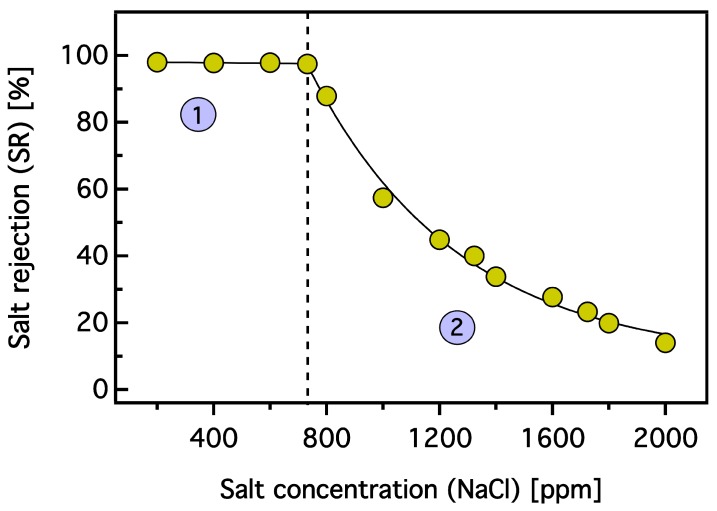
The influence of the salt concentration in the feed solution on the salt rejection, using a GG10k–30 hydrogel. The straight lines are linear and exponential fits to the data in the regime 1 and 2, respectively.

In this sense, we performed a consecutive swelling/deswelling experiment with the best trade-off sample (GG10k–30) to determine the number of consecutive steps required to achieve a complete salt rejection (Figure 16). An initial salt concentration of 2000 ppm can be totally depleted only after four desalination steps. Every measurement step was carried out using new dry hydrogel material and the feed saline water was prepared with respect to the salt rejection measured in the previous step. After the optimisation of the working material, which is the only requirement for our proposed desalination to be controlled, the efficiency of the desalination process was also estimated. The efficiency was calculated from the fractions of water recovery and the salt concentration reduction in each step of the 4-stage process, for a total salt depletion from the initially 2 g L${}^{-1}$ brine solution. The value is found to be ∼0.4 kg hydrogel per square metre freshwater using the best trade-off hydrogel material. Since the salt rejection is a function of the respective hydrogel structure and composition, we measured it for the same type of hydrogel. In this context, salt concentration of the feed solution as a function of salt rejection was also determined using the same hydrogel material. As shown in Figure 17, the influence of the salt concentration on the desalination process using a GG10k–30 hydrogel can be separated into two regimes. In the first regime (from 200 up to 750 ppm), the salt is almost totally rejected and the salt rejection is independent of the input concentration because the ionic content (the charge concentration of the polyelectrolyte) in the gel phase is higher than outside. In contrast, the salt rejection decreases exponentially in the second regime as the salt concentration in the feed solution exceeds a critical value of about 750 ppm of NaCl. However, this critical value is dependent on the effective charge density of the polyelectrolyte, which is correlated with the network structure on a given polymer volume fraction in the respective swollen state.

**Figure 18:**
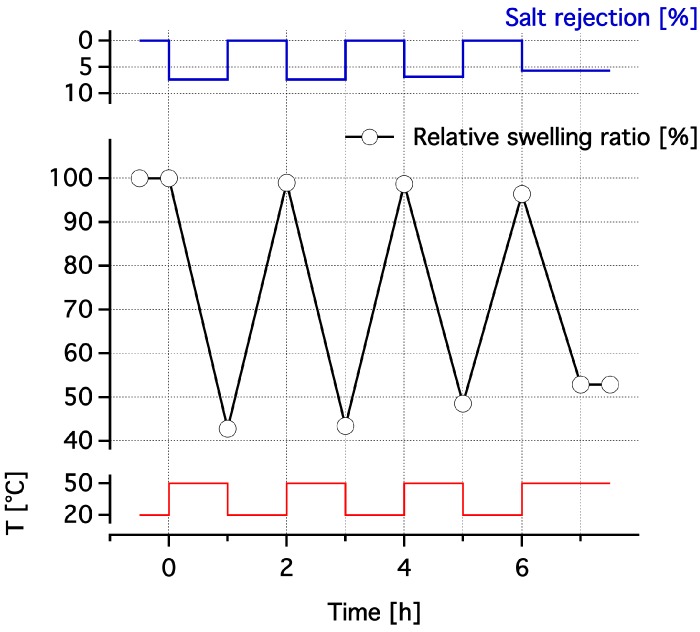
Swelling/dewatering cycles of GG10K–20 gel (powder form) in response to temperature changes for 2 h cycles between 20 and 50 ${}^{\circ }$C in brine solution of 2 g L${}^{-1}$ NaCl, and the corresponding salt rejection for each cycle.

From an application point of view, it is also important to study the reversibility of the desalination process. We evaluated the reversibility of the swelling ratio and the salt rejection on temperature cycles between 20 and 50 ${}^{\circ }$C over 2 h for a GG10k–20 gel (Figure 18). After each cycle, the salt depleted water was taken out from the mixture and the completely dried gel at ambient conditions was reused for the next cycle. It can be observed that both the dewatering level and the salt rejection start to decrease after the first two cycles, the total water content decreases slightly, whereas more water stays in upon heating.albeit the relative swelling ratio is nearly constant, i.e., dewatering is slower than swelling over the time scale. This may be attributed to the formation of denser or more stable hydrated polymer layers within the hydrogel network resulting from the reorientation of the grafted PAAc chains upon each cycle. The reduced percentage of the recovered water could be also caused by the delayed formation of hydrophobic nuclei during gel shrinking.

## 4. Conclusions

The total world water demand is predicted to be a global interdisciplinary research challenge. This need is primarily driven by rapid growth of population and industries, which leads to high water consumption. Despite the vast abundance of water, a lot of effort has to be done for human consumption or other beneficial purpose. Advanced technologies for water purification and desalination are currently in use. Most of these technologies such as reverse osmosis and depend on nonrenewable traditional fossil fuels, which are subject to a rapid decrease with increasing world population. Therefore, the search for a viable alternative technology is becoming important from an economic standpoint. In this research, we reported on the development of polymer-based hydrogels for salty water desalination. These hydrogels were synthesised by a combination of grafted polyelectrolyte and a thermally responsive polymer with different grafted chain content and chain length. We have shown that the efficiency of this desalination process strongly depends on their composition. In particular, altering the grafted PAAc content has a major influence on swelling/dewatering behaviour as well as the salt expelling ability of the hydrogels. The recovered water in the dewatering step decreases and meanwhile the salt rejection increases as the PAAc content increases. In contrast, hydrogels with the same chemical composition but different PAAc-grafted chain length reveal a higher swelling capacity as the chain length increases, while the percentage of released water increases only slightly as a function of the PAAc-grafted chain length. It was found that the performance of a material with 30 wt % of grafted PAAc and a chain length of 10 kD is the best trade-off with respect to salt removal and and water recovery for the proposed desalination experiment. Using this material, the total salt depletion of 2 g L${}^{-1}$ brine can be achieved after four desalination steps with an efficiency of ∼0.4 kg hydrogel per square metre freshwater. However, other aspects that are of major importance prior to real application still have to be addressed including real brackish water samples, which may contain divalent ions, heavy metals and biocontaminants.

## Figures and Tables

**Table 1:** Composition, experimental conditions, and analysis of the synthesised macro-RAFT agents PAAc-TTC in 1,4-dioxane and their molecular characteristics ${}^{a}$.

Expt.	Macro-RAFT Agent	${\left[\mathrm{AAc}\right]}_{0}$ (mol L${}^{-1}$)	${\left[\mathrm{AAc}\right]}_{0}/$ ${\left[\mathrm{TCCA}\right]}_{0}$	Time (min)	Conv. ${}^{b}$ (mol %)	$M_{n}^{\mathrm{th}}$ (g mol${}^{-1}$)	$M_{n}^{\mathrm{NMR}}$ (g mol${}^{-1}$)
E 1	PAAc3K	1.50	90	80	45.5	3320	3560
E 2	PAAc5K	2.07	130	80	48.8	4930	5071
E 3	PAAc10K	3.33	233	80	56.4	9830	10551

${}^{a}$ The polymerisations were performed with a [TTCA]_0_/ [ACPA]_0_ ratio of 10; ${}^{b}$ Monomer conversion determined by ^1^H-NMR.

**Table 2:** Composition and experimental conditions for the synthesis of PNIPAAm-*g*-PAAc networks by RAFT polymerisation ${}^{a}$.

Expt.	Gel Code ${}^{b}$	PAAc-TTC (wt %)	NIPAAm (wt %)	NIPAAm Conv. ${}^{c}$ (wt %)	PAAc-TTC Content (wt %)
G1	GG3K–20	20	80	98.4	20.3
G2	GG3K–30	30	70	96.2	31.1
G3	GG3K–40	40	60	94.7	42.2
G4	GG3K–50	50	50	–	–
G5	GG5K–20	20	80	98.0	20.4
G6	GG5K–30	30	70	97.2	30.8
G7	GG5K–40	40	60	93.5	42.6
G8	GG5K–50	50	50	–	–
G9	GG10K–20	20	80	96.8	20.6
G10	GG10K–30	30	70	95.1	31.5
G11	GG10K–40	40	60	93.4	42.6
G12	GG10K–50	50	50	–	–

${}^{a}$ The polymerisations were performed in ethanol (3 mL) at 70 ${}^{\circ }$C with a ratio of [ACAP]_0_/[PAAc-TTC]_0_ = 1 mol % and [MBAm] = 0.05 g; ${}^{b}$ The nomenclature of the hydrogels (gel code) was given based on the chain length and the content of PAAc-TTC, respectively, while GG represented the abbreviation of graft gel. ${}^{c}$ Monomer weight conversion determined by gravimetric analysis.

## Supplementary Materials

The following are available online at http://www.mdpi.com/2073-4360/10/6/567/s1, Characterisation of the poly(acrylic acid) macromolecular RAFT agent (PAA-TTC) by ^1^H-NMR and FTIR; characterisation of hydrogels by FTIR; time dependence of the hydrogel swelling; storage modulus ($G^{\prime }$) and loss tangent (tanδ) as a function of angular frequency (ω) of all hydrogels; and the performance of hydrogels in terms of total relative performance.
