# Supplementary material for: On the Potential of Using Dual-Function Hydrogels for Brackish Water Desalination
Source: Polymers (Basel). 2018 May 23;10(6):567. doi: 10.3390/polym10060567 (PMC6403859; doi:10.3390/polym10060567)
Supplement: Supplementary file 1 [file polymers-10-00567-s001.pdf]

Supplementary Materials for

On the potential of using dual-function hydrogels for brackish water desalination

Wael Ali [1,2,4], Beate Gebert [2], Sedakat Altinpinar [1], Thomas Mayer-Gall [1,2], Mathias Ulbricht [3], Jochen S. Gutmann*[1,2] and Karlheinz Graf[4]

[1] Physikalische Chemie and CENIDE (Center for Nanointegration), Universitï£¡t Duisburg-Essen, Universitï£¡tsstr. 2, 45141 Essen, Germany
[2] Deutsches Textilforschungszentrum Nord-West gGmbH, Adlerstr. 1, 47798 Krefeld, Germany
[3] Technische Chemie II and CENIDE (Center for Nanointegration), Universitï£¡t Duisburg-Essen, Universitï£¡tsstr. 5, 45141 Essen, Germany
[4] Physikalische Chemie, Hochschule Niederrhein, Adlerstr. 32, 47798 Krefeld, Germany

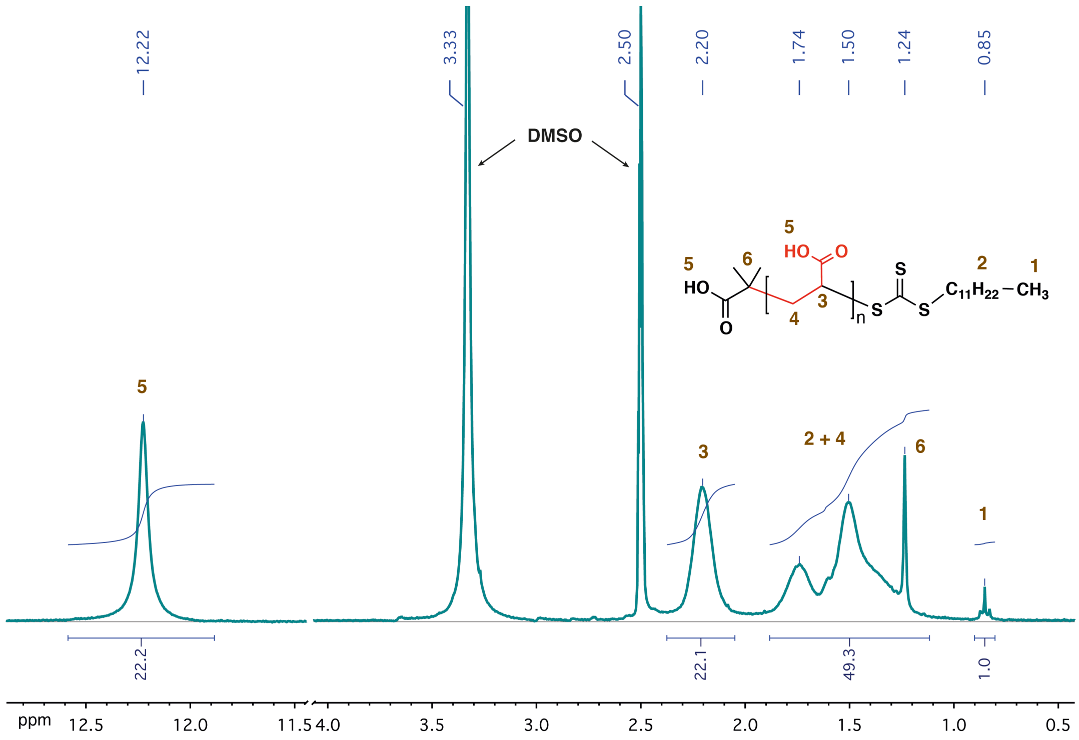

Figure S 1 Partial ^{1}H-NMR spectrum of poly(acrylic acid) macromolecular RAFT agent (PAA-TTC).

-

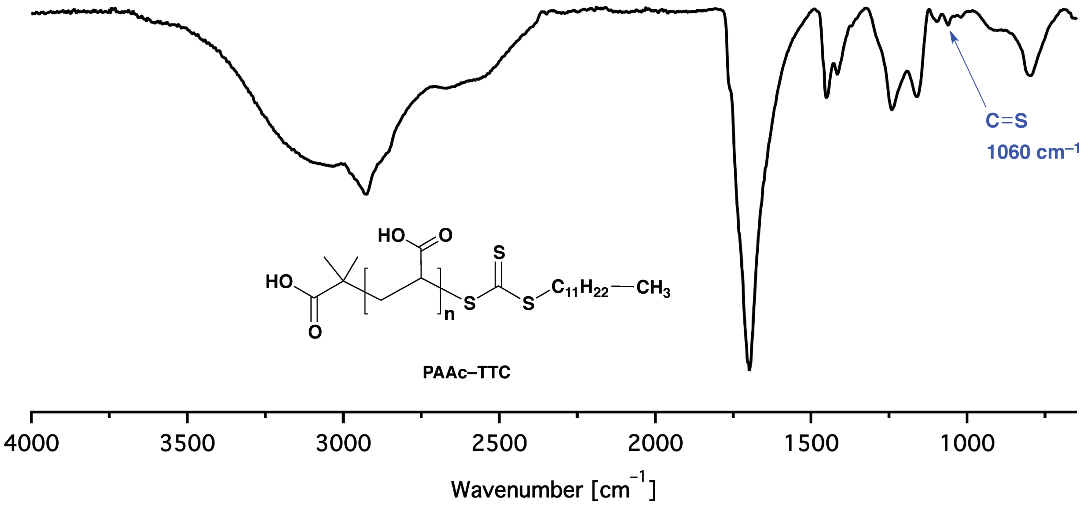

Figure S 2 FTIR spectrum of poly(acrylic acid) macromolecular RAFT agent (PAA-TTC).

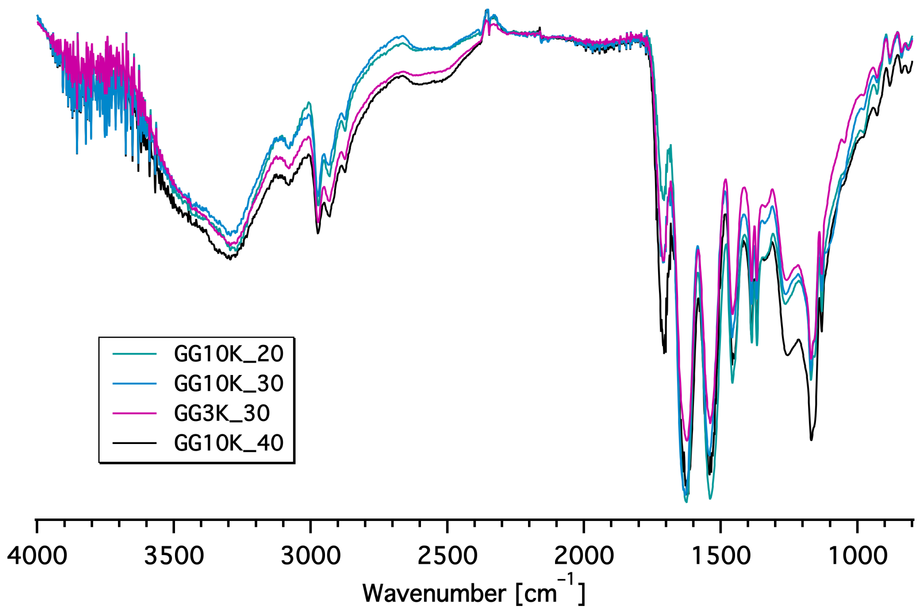

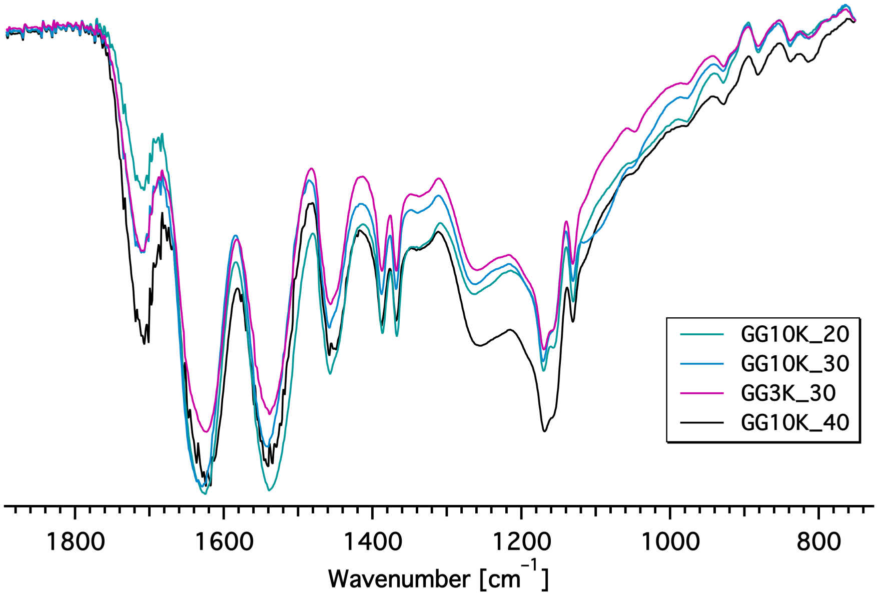

Figure S 3 FTIR spectra of hydrogels investigated in detail in this work.

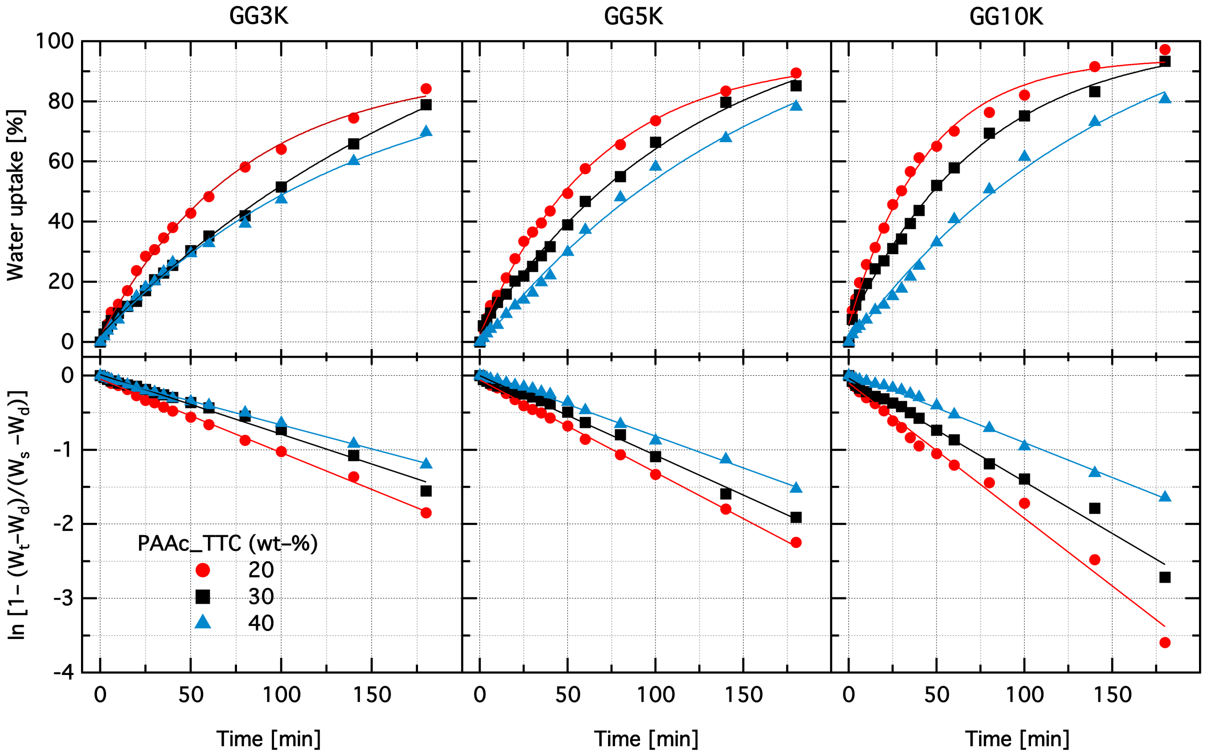

Figure S 4 Plots of water uptake as a function of swelling time in deionised water (DI) at room temperature (23 °C) (*upper plots*) and a semilogarithmic plots as first-order rate analysis was applied to the time dependence of the swelling (*lower plots*) to obtain the swelling rate constant, from which the half-swelling time ($t_{1/2}$) was obtained. The sample shape and size were constant for all hydrogels (cylinder, 5 mm in diameter and 2 mm in thickness).

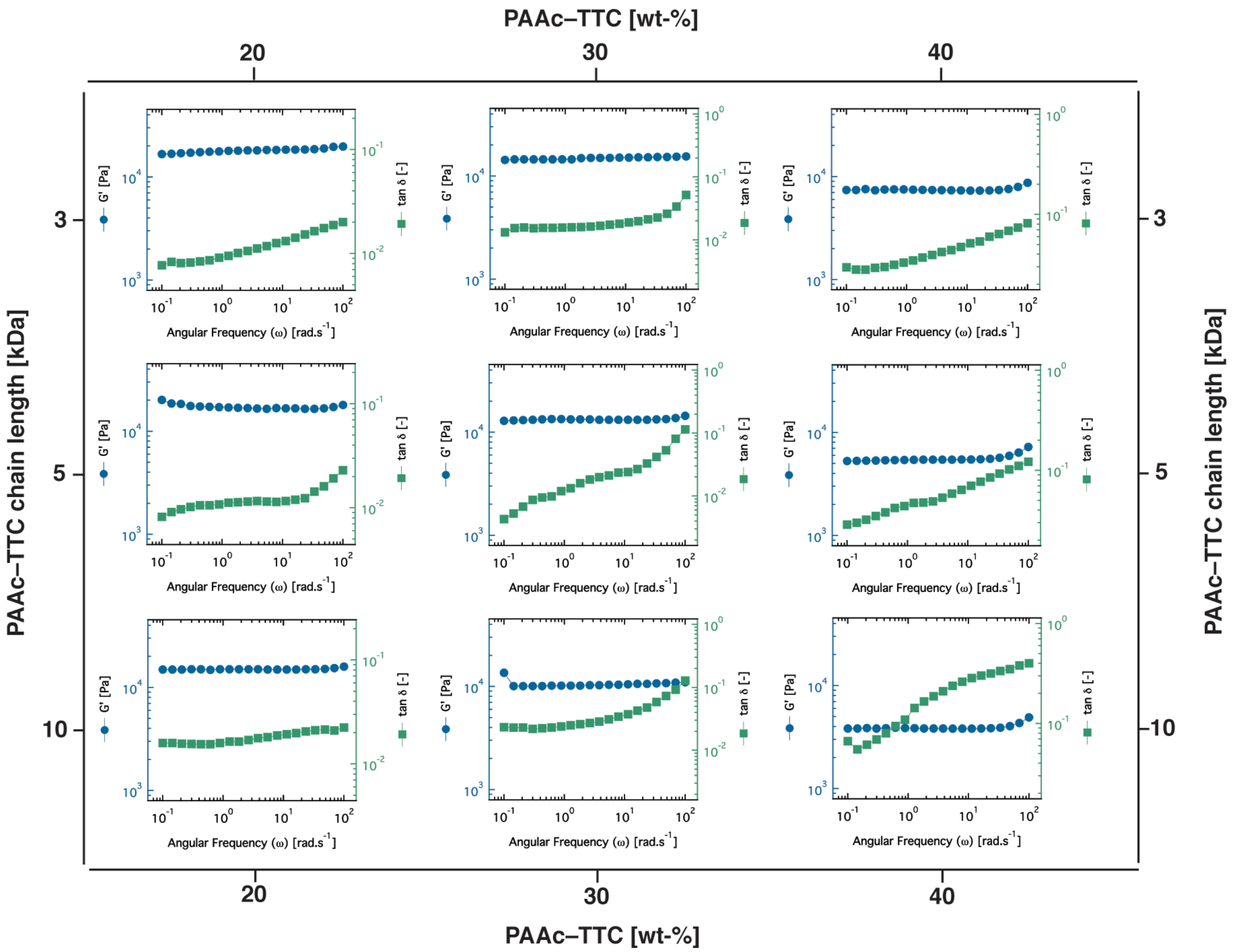

Figure S 5 Storage modulus (G') and loss tangent ($tan\delta$) as a function of angular frequency (ω) of all hydrogels.

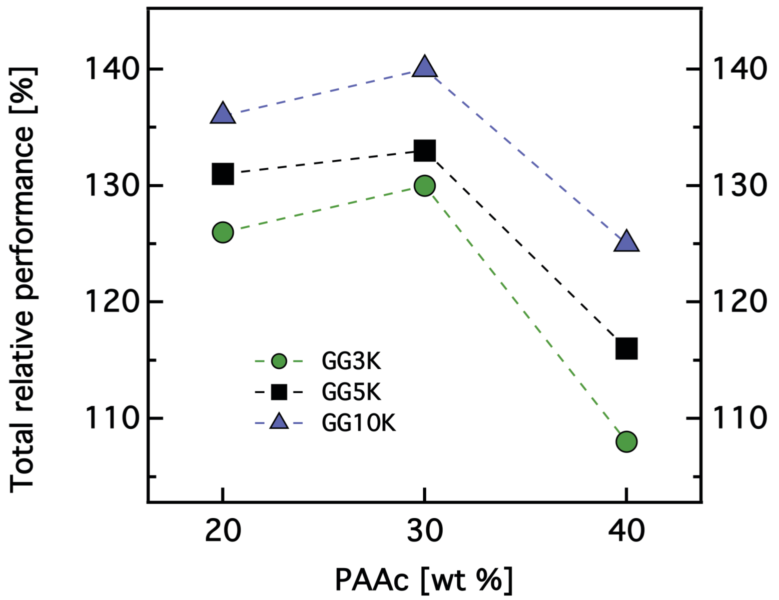

Figure S 6 The performance of hydrogels in terms of total relative performance (water recovery and salt rejection) as a function of grafted-PAAc content. The dashed lines are a guide to eye.
